# The Predation Strategy of *Myxococcus xanthus*

**DOI:** 10.3389/fmicb.2020.00002

**Published:** 2020-01-14

**Authors:** Susanne Thiery, Christine Kaimer

**Affiliations:** Department of Biology and Biotechnology, Ruhr University Bochum, Bochum, Germany

**Keywords:** bacterial soil communities, protein secretion system, outer membrane vesicle, myxovirescin, gliding motility

## Abstract

Myxobacteria are ubiquitous in soil environments. They display a complex life cycle: vegetatively growing cells coordinate their motility to form multicellular swarms, which upon starvation aggregate into large fruiting bodies where cells differentiate into spores. In addition to growing as saprophytes, Myxobacteria are predators that actively kill bacteria of other species to consume their biomass. In this review, we summarize research on the predation behavior of the model myxobacterium *Myxococcus xanthus,* which can access nutrients from a broad spectrum of microorganisms. *M. xanthus* displays an epibiotic predation strategy, i.e., it induces prey lysis from the outside and feeds on the released biomass. This predatory behavior encompasses various processes: Gliding motility and induced cell reversals allow *M. xanthus* to encounter prey and to remain within the area to sweep up its biomass, which causes the characteristic “rippling” of preying populations. Antibiotics and secreted bacteriolytic enzymes appear to be important predation factors, which are possibly targeted to prey cells with the aid of outer membrane vesicles. However, certain bacteria protect themselves from *M. xanthus* predation by forming mechanical barriers, such as biofilms and mucoid colonies, or by secreting antibiotics. Further understanding the molecular mechanisms that mediate myxobacterial predation will offer fascinating insight into the reciprocal relationships of bacteria in complex communities, and might spur application-oriented research on the development of novel antibacterial strategies.

## Introduction

Predatory bacteria are found in different phyla and apply different mechanisms to kill bacteria of other species and consume their biomass (Jurkevitch, 2007). Endobiotic predators, like the *Bdellovibrio* species, enter the periplasm of a prey cell, utilize its biomass to multiply and lyse the prey cell from within (Sockett, 2009). In contrast, epibiotic predators induce prey lysis from the outside and feed on the released biomass. Epibiotic predation has been described for different proteobacteria, such as *Ensifer adhaerens* and *Micavibrio admirandus* (*α*-proteobacteria), *Cupriavidus necator* (*β*-proteobacteria), *Lysobacter* sp. (*γ*-proteobacteria) and the Myxobacteria (*δ*-proteobacteria) (Jurkevitch, 2007; Pérez et al., 2016).

**Figure 1:**
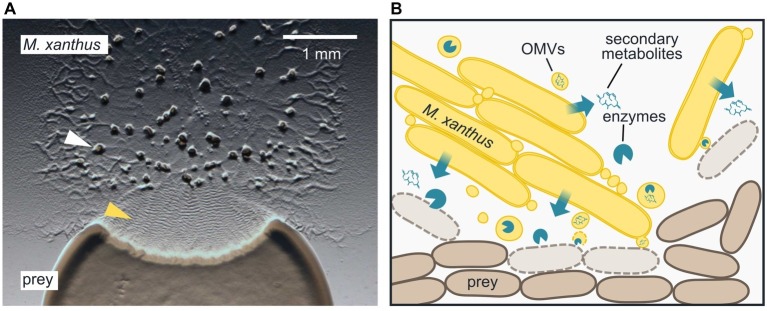
Epibiotic predation by the myxobacterium *Myxococcus xanthus*. **(A)** *M. xanthus* cells that are placed next to *E. coli* on a CF agar plate, which only provides a minimal amount of nutrients, expand radially using gliding motility, enter the prey colony, and lyse prey cells. Multicellular fruiting bodies (white arrowhead), in which *M. xanthus* cells differentiate into spores, start to emerge near the inoculation spot. Preying *M. xanthus* induces regular cell reversals, which appear as macroscopic ripples within the prey area (yellow arrowhead). The image was taken 2 days after the initial inoculation of predator and prey. **(B)** *M. xanthus* secretes hydrolytic enzymes and secondary metabolites, which presumably kill and degrade prey cells for biomass acquisition. Outer membrane vesicles (OMVs) may contribute to the delivery of these lytic factors. *M. xanthus* cells typically move and prey in large clusters, but also individual cells are able to induce prey cell lysis.

Myxobacteria are ubiquitous in soil and known for their elaborate multicellular behaviors (Keane and Berleman, 2016; Muñoz-Dorado et al., 2016): single cells form large clusters that move slowly along surfaces and secrete enzymes at high concentrations to access nutrients. When nutrients become scarce, cells coordinate their motility and aggregate into complex multicellular structures, called fruiting bodies, where they differentiate into spores (Figure 1A, white arrowhead). In this mini-review, we summarize our current understanding of the epibiotic predation strategy displayed by the model myxobacterium *Myxococcus xanthus.*

Predation is a facultative behavior of *M. xanthus,* since it can also grow saprophytically by degrading dead biomass derived from plants or microorganisms (Shimkets, 1984). Its preferred energy and carbon source are amino acids and lipids, which are catabolized *via* various pathways. Moreover, *M. xanthus* lacks the biosynthetic pathways for valine, leucine, and isoleucine and thus relies on external sources for these branched amino acids (Curtis and Shimkets, 2008). Comparative genome analysis revealed that genes associated with secondary metabolites, digestive enzymes, protein secretion, and TonB-dependent receptors/metal transport, which may be utilized for killing other bacteria and consuming their biomass, are overrepresented in *M. xanthus* (Goldman et al., 2006; Karlin et al., 2006; Korp et al., 2016).

Compared to other bacterial predators, which display a limited prey spectrum, *M. xanthus* is a generalist that is able to feed on a broad range of bacteria and some fungi: It has been observed to prey on soil bacteria (Rosenberg and Varon, 1984; Morgan et al., 2010; Mendes-Soares and Velicer, 2013), including plant pathogens (Pham et al., 2005), on cyanobacteria (Shilo, 1970), clinically relevant species, such as *Pseudomonas aeruginosa* and *Staphylococcus aureus* (Müller et al., 2014; Livingstone et al., 2017), yeasts (Berleman et al., 2006; Livingstone et al., 2017), and other fungi (Bull et al., 2002). Comparative analysis of predation performance indicates that, under laboratory conditions, most prey species significantly support *M. xanthus* growth (Morgan et al., 2010; Mendes-Soares and Velicer, 2013; Livingstone et al., 2017). Considering the broad spectrum of prey that can be utilized by *M. xanthus*, it is likely that different molecular mechanisms, acting either in isolation or synergistically, are required to prey on different species. Moreover, numerous studies addressing different aspects of *M. xanthus* predation have corroborated that predation is a complex, multilayered process that requires a combination of different traits: prey cells are encountered, recognized, and lysed, while the predator remains unharmed. Biomass released from prey is degraded, taken up, and metabolized. These processes can require individuals or a large number of predator cells and may evoke a specific response by the prey.

## Gliding Motility Allows Frequent Prey Encounters

Regardless of the prey species used, *M. xanthus* must be in close proximity to prey cells in order to induce their lysis and to benefit from their biomass (Figure 1B; McBride and Zusman, 1996; Berleman and Kirby, 2009; Muñoz-Dorado et al., 2016). *M. xanthus* encounters prey cells by gliding slowly on surfaces, powered by two motility mechanisms (Mercier and Mignot, 2016; Schumacher and Søgaard-Andersen, 2017): polar type IV pili are extended and retracted to move by “social” S-motility, where cells typically form large clusters that are embedded in exopolysaccharides. Alternatively, *M. xanthus* cells can move as individuals, which are powered by motility engines that are laterally embedded in the cell envelope (“adventurous” A-motility). The S- and A-motility mechanisms were both shown to contribute to efficient predation (Pham et al., 2005; Hillesland et al., 2007; Pérez et al., 2014).

By using time-lapse microscopy, it was demonstrated that an individual *M. xanthus* cell is able to induce lysis of an *E. coli* cell (Figure 1B; McBride and Zusman, 1996; Zhang et al., 2019). Moreover, the *M. xanthus* cell was observed to change its behavior upon contact with *E. coli*, by halting next to a prey cell (Zhang et al., 2019) and/or repeatedly reversing its direction of movement (McBride and Zusman, 1996). This presumably allows *M. xanthus* to remain within the area and ensure lysis of all available prey cells. Cell reversals in *M. xanthus* are regulated by a specialized chemosensory system, the *frz* pathway (Kaimer et al., 2012), and mutation of key regulators reduces predation efficiency (Pham et al., 2005; Berleman et al., 2008). Regulated reversals are also essential for the characteristic rippling pattern, which can be observed in preying *M. xanthus* populations: upon entering and lysing a prey colony, *M. xanthus* cells synchronize their motility and reverse in oscillating waves (Figure 1A, yellow arrowhead) (Reichenbach, 1966; Berleman et al., 2006, 2008). It was shown, both experimentally and by computational simulation, that rippling in presence of prey increases the rate of colony expansion by the predator (Berleman et al., 2008; Zhang et al., 2012). At the same time, rippling reduces the mean square displacement of individual cells, which would allow the predator cells to remain within the prey area longer (Zhang et al., 2012).

Overall, regulated cell motility determines how frequently *M. xanthus* encounters prey, and therefore mutations in the respective genes reduce predation efficiency. However, the ability to kill and consume prey cells is not affected, as motility mutants are still able to lyse and grow on prey (Berleman and Kirby, 2009).

## How are Prey Cells Lysed and Consumed?

According to our current understanding, *M. xanthus* uses a combination of secondary metabolites and secreted enzymes to kill and lyse prey cells, and to consume their biomass (Figure 1B; Keane and Berleman, 2016; Muñoz-Dorado et al., 2016; Pérez et al., 2016). *M. xanthus* encodes for 24 biosythetic clusters that produce secondary metabolites. In addition to several unknown products, these clusters synthesize pigments, siderophores, bacteriocins, and antibiotics that target bacteria or fungi (Korp et al., 2016). For two antibiotics, myxovirescin A and myxoprincomide, a role in predation has been demonstrated. Myxoprincomide is a linear peptide of nine amino acids that is synthesized by a hybrid biosynthetic module of non-ribosomal peptide/polyketide synthases (Cortina et al., 2012), but its mode of action remains unknown. Lack of myxoprincomide reduces *M. xanthus* growth on *B. subtilis* and increases the number of surviving prey cells (Müller et al., 2016). However, myxoprincomide is not required for growth of *M. xanthus* on *E. coli*, and also not for growth on a *B. subtilis* mutant that is unable to produce the antibiotic bacillaene (Müller et al., 2014, 2016). It therefore does not appear to be a general killing factor, but might rather be required to specifically counteract antibiotics that are produced by prey in response to *M. xanthus* (Müller et al., 2016).

Myxovirescin A (or “TA”) is a bactericidal antibiotic, which targets signal peptidase II (LspA) and thereby inhibits lipoprotein maturation, leading to the formation of lethal cross-links between the cell wall and the inner membrane in growing cells (Rosenberg and Varon, 1984; Xiao et al., 2012). In co-culture experiments, significantly more *E. coli* cells survive in the presence of a myxovirescin A mutant compared to *M. xanthus* wild type, which implies a role for myxovirescin A in prey killing (Xiao et al., 2011). However, lack of myxovirescin makes no difference for predation under nutrient-free conditions, i.e., when prey cells are not actively growing. The resulting hypothesis is that bactericidal antibiotics, such as myxovirescin A, are secreted by *M. xanthus* to neutralize prey metabolism, which might facilitate the subsequent degradation of prey biomass by hydrolytic enzymes (Xiao et al., 2011). Obviously, prey killing can be accomplished by other secondary metabolites or additional mechanisms. The production of myxovirescin is controlled by the alternative sigma factor σ^54^ in conjunction with specific enhancer binding proteins (EBPs) (Volz et al., 2012). σ^54^-dependent regulators are abundant in *M. xanthus* (Goldman et al., 2006; Karlin et al., 2006) and regulate the expression of many secondary metabolites and proteins, mainly during development (e.g., Jelsbak et al., 2005). Transcriptional control by σ^54^/EBPs might therefore coordinate secondary metabolite production, development, and predation (Volz et al., 2012).

The production and secretion of hydrolytic proteins by *M. xanthus* is well documented (Rosenberg and Varon, 1984): bacteriolytic proteins with amidase and glucosaminidase activity have been isolated from *M. xanthus* culture supernatant early on (Hart and Zahler, 1966; Sudo and Dworkin, 1972). Sequencing of the *M. xanthus* genome revealed several proteins with an annotated function related to peptidoglycan degradation, as well as numerous secreted enzymes with putative proteolytic activity (Goldman et al., 2006). Proteases and peptidases, such as MepA (Berleman et al., 2014), presumably contribute to predation by degrading proteins that are released from prey cells. More importantly, enzymes with peptidoglycan-degrading activity could potentially serve as killing factors that induce prey cell lysis. Recently, GluM, an outer membrane *β*-1,6-glucanase of the myxobacterium *Corallococcus*, was shown to be required for lysing the chitinous cell wall of certain fungi (Li et al., 2019). The homologous protein in *M. xanthus*, Oar, has glucanase activity as well (Li et al., 2019), but was independently shown to be involved in exporting a protease that is required for induction of the cell differentiation program (Gómez-Santos et al., 2019). The possibility of a dual role of Oar in the predation of fungi and the regulation of development remains to be investigated, but these observations emphasize a putative role of cell wall-lytic proteins as killing factors in myxobacterial predation. However, while the expression and secretion of bacteriolytic enzymes by *M. xanthus* is unquestionable, predation factors that might specifically mediate the lysis of prey cells have not been identified, yet.

## The Delivery of Predation Factors

The requirement for close proximity to induce prey lysis implies that the delivery of killing factors by *M. xanthus* is locally restricted. For protein effectors, this could be achieved by targeted secretion *via* protein secretion systems of the types II, III, IV or VI, which are used in other bacteria to deliver lethal effectors in the proximity or directly into prokaryotic or eukaryotic cells (Galán and Waksman, 2018). The *M. xanthus* genome encodes for protein secretion systems of type II, III (incomplete), and VI (Konovalova et al., 2010). The type VI secretion system (T6SS) was shown to mediate antagonism between different kin of myxobacteria, and even between physiologically challenged siblings of the same kin (Troselj et al., 2018). One effector delivered *via* the T6SS is the toxin MXAN_0050, a nuclease, against which the secreting cell protects itself by a concomitantly expressed anti-toxin, MXAN_0049 (Gong et al., 2018). However, it has not been reported, whether the T6SS and MXAN_0050 contribute to killing of other species for biomass acquisition during predation. A recent study reports that the survival of *E. coli* in prolonged co-culture with *M. xanthus* correlates with mutation of the *E. coli* outer membrane protease, OmpT (Nair et al., 2019). This led to the intriguing suggestion that pre-lytic factors secreted by *M. xanthus* might be activated by the prey itself, indicating a putative mechanism to limit lysis to the prey cells.

Another mode of protein secretion and delivery may be provided by outer membrane vesicles (OMVs, Figure 1B), which are produced by many Gram-negative bacteria (Palsdottir et al., 2009; Kulp and Kuehn, 2010). Indeed, *M. xanthus* OMVs were shown to contain lytic components and to be involved in predation (Evans et al., 2012; Berleman et al., 2014). OMVs emerge after fission from the secreting cell and are released into the environment. When OMVs reach a recipient cell *via* diffusion, they could deliver their cargo by fusing with the outer membrane of Gram-negative bacteria, or by disintegrating next to a Gram-positive cell (Whitworth, 2011). The packaging of lytic compounds into a vesicle instead of freely secreting them into the extracellular space might provide several advantages: The lytic compounds remain at high local concentration, and the slow passive diffusion of a large vesicle increases the likelihood of a secreting cell to be in close proximity for nutrient uptake after prey killing, reducing the risk of exploitation by non-secreting cells (Whitworth, 2011). Moreover, delivering a cocktail of various lytic factors would increase efficiency and prevent resistance formation by prey (Berleman et al., 2014). With OMVs, membrane-associated lytic components could also be transported to the prey cell, and soluble secreted cargo would be protected from the environment (Kulp and Kuehn, 2010).

The proteome of *M. xanthus* OMVs has been shown to differ from that of the periplasm and outer membrane, hinting to the presence of a dedicated protein sorting mechanism to load OMVs (Kahnt et al., 2010; Evans et al., 2012; Berleman et al., 2014; Whitworth et al., 2015). OMVs contain several proteins with predicted hydrolytic functions, as well as antibiotics, such as myxovirescin A and the antifungal myxalamide (Kahnt et al., 2010; Evans et al., 2012; Berleman et al., 2014). Indeed, the lytic potential of isolated *M. xanthus* OMVs toward *E. coli* was experimentally demonstrated (Evans et al., 2012; Remis et al., 2014). However, as in the case of freely secreted enzymes, it is not clear, which of the OMV cargo components mediate prey killing or just degradation of dead biomass. Furthermore, the lytic effect of *M. xanthus* OMVs toward Gram-negative bacteria other than *E. coli* or Gram-positive bacteria has not been been demonstrated, so far. In multicellular swarms, individual *M. xanthus* cells were observed to be connected *via* a network of membrane tubes and OMV chains. It was hypothesized that these appendices may play a role for inter-cellular signaling and outer membrane exchange among myxobacteria (Pathak et al., 2012; Ducret et al., 2013; Remis et al., 2014). However, membrane tubes were also observed under experimental conditions that prevent outer membrane exchange, which suggests that both processes are functionally separate (Wei et al., 2014). It remains to be investigated, how outer membrane structures within a myxococcal swarm relate to the proposed function of OMVs in the delivery of lytic factors during predation, and how *M. xanthus* cells might protect themselves against their lytic cargo.

## Regulatory Mechanisms and Prey Counteractions

The synthesis and release of putative predation-specific lytic factors by *M. xanthus* consumes energy and resources, and can only be productive when prey cells are available. A transcriptome study singled out only a few *M. xanthus* genes that are differentially expressed when starved cells are supplied with live *E. coli* prey, suggesting the constitutive expression of the predatory arsenal under the tested conditions (Livingstone et al., 2018). However, *M. xanthus* gene transcription did respond both to the addition of pre-killed bacteria and of hydrolyzed peptides. These observations indicate that lysed bacteria, but not live prey cells, are perceived as nutrients (Livingstone et al., 2018). If the production of lytic factors is not regulated on the level of transcription, their release from the cell might be triggered upon prey encounter to reduce the loss of costly molecules (Berleman and Kirby, 2009). Indeed, individual *M. xanthus* cells alter their motility behavior when in contact with live prey, but not with dead cells (McBride and Zusman, 1996; Zhang et al., 2019). This indicates that *M. xanthus* can recognize cells of other bacterial species and can discriminate live from dead cells, but the underlying molecular mechanisms are unknown. When competing with other myxobacterial kin groups, *M. xanthus* is able to identify its own kin and eliminate cells of relatives (Vos and Velicer, 2009). This form of self/non-self discrimination is mediated by a modular surface receptor, TraA, that differs among *M. xanthus* kin groups (Pathak et al., 2013; Vassallo et al., 2017; Vassallo and Wall, 2019). However, a possible function for this system during predation of other bacterial species has not been described, yet.

While the recognition and response to live prey cells remains elusive, it is clear that *M. xanthus* can sense and react to the presence of lysed prey: peptidoglycan (or its break-down products) induce coordinated cell reversals that induce rippling patterns (Shimkets and Kaiser, 1982; Berleman et al., 2006). Although this behavior has been termed “predataxis” and requires the *frz* chemosensory pathway (Berleman et al., 2008), it does not represent a directed, chemotactic movement of *M. xanthus* toward prey. In fact, several studies have shown that *M. xanthus* is not chemotactic toward prey (Dworkin, 1983; Shi and Zusman, 1993), and it remains controversial, whether *M. xanthus* shows chemotactic behaviors in general (Dworkin and Eide, 1983; Shi et al., 1993; Mercier and Mignot, 2016). However, Shi and Zusman observed chemotactic behavior of *E. coli* prey toward *M. xanthus* when conducting predation assays under conditions where both *M. xanthus* and *E. coli* are motile (Shi and Zusman, 1993). This led to the striking proposal that *M. xanthus* releases chemoattractants as a primitive form of prey ensnarement, which still awaits further investigation.

Several prey bacteria have been observed to induce specific counteractions to protect themselves against predation by *M. xanthus*, with the predominant mechanisms being the formation of a mechanical barrier or the secretion of antibiotics. For example, *Bacillus subtilis* builds biofilm megastructures, which are filled with spores that cannot be attacked by *M. xanthus* (Müller et al., 2015). Also, biofilm-forming *E. coli* with a matrix of curli are less sensitive to predation by *M. xanthus* than non-biofilm producing mutants (DePas et al., 2014). Recently, co-evolution experiments with *E. coli* and *M. xanthus* demonstrated that predation, in fact, selects toward a mucoid prey phenotype (Nair et al., 2019). Similarly, a galactoglucane-producing *Sinorhizobium meliloti* strain is more resistant toward *M. xanthus* predation compared to the non-mucoid mutant (Pérez et al., 2014). The exact mechanism behind the resistance provided by biofilm barriers is not clear, but they might simply obstruct access of predator cells and their lytic factors to prey. Another protective strategy is the production of antibiotics: *B. subtilis* was reported to escape killing by *M. xanthus* by production of the antimicrobial peptide bacillaene (Müller et al., 2014, 2015). Although this compound does not impede predator growth, it seems to transiently inhibit predation, providing *B. subtilis* with enough time to differentiate into predation-resistant spores (Müller et al., 2014). *Streptomyces coelicolor* produces an antibiotic, actinorhodin, upon co-cultivation with *M. xanthus*, which however does not confer a survival advantage (Pérez et al., 2011). Recently, an enzyme released by *Bacillus licheniformis* was found to glycosylate myxovirescin A, which inactivates the antibiotic and renders the producing strain less susceptible to predation by *M. xanthus* (Wang et al., 2019).

## Predation as a Multicellular Behavior

The *M. xanthus* life cycle is largely directed to maintain multicellular interactions, for example manifested in social motility and coordinated cell differentiation (Dworkin and Bonner, 1972). Vegetative growth and predation have been discussed in the context of multicellularity, since Rosenberg et al. observed that the growth rate of *M. xanthus* growing in liquid culture increases depending on cell density, and suggested that nutrient acquisition during predation might involve cooperative behavior similar to a “wolf pack,” where the cooperation between individuals with specific tasks enhances the efficiency of the group: hydrolytic enzymes to degrade prey biomass are secreted by the members of a *M. xanthus* swarm as common goods and increase in concentration with the number of secreting cells, allowing the efficient degradation of (prey) biomass (Rosenberg et al., 1977). This indicated that predation by a *M. xanthus* swarm might be more efficient than by individual cells. Indeed, Zhang et al. recently reported that individual *M. xanthus* cells most often leave *E. coli* prey they have killed without degrading the biomass, presumably because they do not produce a sufficient amount of degradative enzymes (Zhang et al., 2019).

The “wolf pack” analogy has been used frequently to describe and discuss the predation behavior of myxobacteria (Berleman and Kirby, 2009; Keane and Berleman, 2016; Muñoz-Dorado et al., 2016; Pérez et al., 2016). Recently, the concept of wolf pack predation by *M. xanthus* was revisited (Marshall and Whitworth, 2019), and a new interpretation of the *M. xanthus* predation strategy was proposed, which highlights that the secretion of lytic factors and nutrient release are in fact proportionate to cell density. Additional arguments were that the secretion of hydrolytic factors during predation likely is constitutive, and that cell density-dependent nutrient hydrolysis in liquid culture cannot be extrapolated to the native soil habitat of *M. xanthus*, in which diffusion parameters of hydrolytic compounds and predator motility play a different role (Marshall and Whitworth, 2019). Indeed, on a solid agar surface an individual *M. xanthus* cell is able to lyse several *E. coli* cells (McBride and Zusman, 1996). Moreover, transposon mutagenesis revealed that mutants that lack exopolysaccharides, and therefore are unable to form stable multicellular swarms, show increased predation (Müller et al., 2016). These observations may point out the impact of individual *M. xanthus* cells, rather than of EPS-coated swarms, for prey killing.

## Concluding Remarks

Predation behavior of *M. xanthus* is a process of astonishing complexity: various prey are specifically recognized and killed, while the predator remains undamaged. Some prey species actively react by initiating counter-mechanisms, which implies elaborate signaling and regulatory pathways. Therefore, *M. xanthus* predation offers fascinating insights into the interspecies interactions that shape bacterial communities. However, addressing the ecological significance of predation in soil environments will require a more detailed understanding of the molecular mechanisms that mediate predation, namely the recognition and killing of prey. A key goal should be to identify additional predation factors that specifically kill bacteria, rather than degrade dead biomass, and to analyze their mode of action, which may also hold information on resistance formation by prey. Understanding the delivery of killing factors might point toward the mechanisms the predator uses for prey recognition and self-protection. However, dissecting the contribution of individual components might be a difficult task, considering the various different compounds that already have been described to be involved in the process, and which possibly have redundant functions. To address the complexity of predation, it might be useful to analyze the behavior of mutants on different temporal and spatial scales, which discriminate between prey killing and biomass acquisition. Finally, a detailed knowledge of the molecular mechanisms that *M. xanthus* uses to kill various prey species might also spur the development of novel antibacterial strategies to control pathogenic bacteria in medicine or agriculture.

## Author Contributions

All authors listed have made a substantial, direct and intellectual contribution to the work, and approved it for publication.

### Conflict of Interest

The authors declare that the research was conducted in the absence of any commercial or financial relationships that could be construed as a potential conflict of interest.
